# Impact of Surgical Timing on Outcomes in Patients With Acute Cholecystitis: A Systematic Review

**DOI:** 10.7759/cureus.72090

**Published:** 2024-10-22

**Authors:** Ahmed Tabaan Alenezi, Sultan A Bin Jerais, Najla Mohammed Hussein Al Yami, Albatoul Abdulaziz Alluhaida, Azzam Khalid Alharbi, Fatemah Salem Jaber Al Salamah, Fatima Faya M Assiri, Muhammad Tahir E Hayat Mohammed, Fatimah Mohammed Duleem Alqahtani, Mutaz Mohammed Otayf

**Affiliations:** 1 General Surgery, North Medical Tower, Arar, SAU; 2 Medicine and Surgery, Almaarefa University, Ministry of Defense, Riyadh, SAU; 3 Medicine and Surgery, Najran University, Najran, SAU; 4 General Practice, Alrass General Hospital, Alrass, SAU; 5 Medicine and Surgery, Qassim University, Unaizah, SAU; 6 Nursing, Khamis Mushait General Hospital, Abha, SAU; 7 Medicine, Umm Al-Qura University, Makkah, SAU; 8 Surgery, Najran University, Najran, SAU; 9 Medicine and Surgery, King Fahad Central Hospital, Jazan, SAU

**Keywords:** acute cholecystitis, complications, delayed intervention, early intervention, mortality, outcomes, surgical timing

## Abstract

This study aims to conduct a thorough analysis of the existing studies to determine how patients with acute cholecystitis (AC) respond to surgical intervention at different times following their diagnosis. To locate studies that met the inclusion criteria, a thorough computerized search of relevant databases was carried out. A comprehensive search was carried out on PubMed, SCOPUS, Science Direct, Cochrane Library, and Web of Science to locate relevant material. Our data included seven trials with 48,747 patients: 40,955 in the early laparoscopic cholecystectomy (ELC) group and 7,792 in the delayed laparoscopic cholecystectomy (DLC) group. More than half of the participants (27,687, 56.8%) were female. ELC was found to be safe regardless of when symptoms first appeared, challenging previous assumptions that surgery should be delayed during the initial symptomatic period. Mortality rates for ELC were consistently low, ranging from 0% to 3.9% in all groups, while complication rates varied from 3.5% to 12.5% in all groups as well. Although DLC is still considered safe, the likelihood of complications such as bile duct damage and surgical site infections appeared to increase, particularly in patients who underwent surgery more than three days after the diagnosis of cholecystitis. It is important to note that ELC refers to surgery performed within the first three days after diagnosis, while DLC refers to surgery performed after three days. The systematic review reveals that ELC is a secure and successful remedy for sudden cholecystitis, offering superior outcomes compared to DLC. ELC is associated with lower complication rates, reduced hospital stays, and minimal mortality, suggesting it should be the preferred approach in most cases (performing the surgery within the first three days following the diagnosis of AC). While DLC remains a viable option, particularly for certain patient populations, it carries a higher risk of complications and prolonged recovery times.

## Introduction and background

Acute cholecystitis (AC) is a frequent surgical emergency that, if left untreated, can result in considerable morbidity and death. It is characterized by inflammation of the gallbladder, commonly caused by gallstones. The timing of surgical intervention plays a crucial role in determining patient outcomes, including the risk of complications, length of hospital stay, and overall recovery [1].

Traditionally, the management of AC involved early surgical intervention, ideally within the first 24 to 48 hours of diagnosis. This approach aims to minimize complications such as perforation, abscess formation, and sepsis, which can arise as the condition progresses [2]. Studies have demonstrated that patients who undergo surgery during this critical window often experience better outcomes, including fewer instances of open surgery conversion and fewer problems following surgery [3].

Conversely, delayed surgery, defined as procedures performed more than 48 hours after diagnosis, has been associated with higher morbidity rates. This delay generally occurs because of the initial cautious therapy, including intravenous fluids and antibiotics, to control inflammation and stabilize the patient [4]. While this strategy may be clinically justified in certain cases, such as in patients presenting with severe comorbidities, it carries risks that can complicate the eventual surgical procedure. Notably, delays can result in adhesion development and a higher chance of problems, including wound infections and biliary leakage [5].

However, the optimal timing of surgery may also be influenced by individual patient factors, such as the severity of the cholecystitis, age, and underlying medical issues. For example, in individuals who are very old or have a lot of comorbidities, a conservative approach may be preferable, prioritizing medical management until it is deemed safe to proceed with surgery. In these cases, thorough risk assessment and patient monitoring become paramount to prevent deterioration [2].

Even in the group of patients who arrive later, laparoscopic cholecystectomy is still a safe and effective alternative, according to emerging data. Some studies suggest that advanced minimally invasive techniques can yield favorable outcomes even when surgery is performed later than the commonly recommended timeframe. Consequently, the paradigm surrounding surgical timing is gradually shifting, emphasizing a more individualized approach based on risk stratification and patient presentation [6].

The timing of surgery in AC remains a critical determinant of patient outcomes. Understanding the optimal timing of surgery can help clinicians make informed decisions and improve patient care. It's still uncertain when AC sufferers should get surgery, with conflicting evidence supporting both early and delayed surgical intervention. Uncertainty like this might result in inconsistent clinical practice and possibly worse than ideal patient results. This study aims to analyze the effect of surgical scheduling on outcomes in patients with AC by conducting a comprehensive evaluation of the literature.

## Review

Methods

In order to investigate the effect of surgical scheduling on outcomes in patients with AC, this study conducted a systematic review utilizing the Preferred Reporting Items for Systematic Reviews and Meta-Analyses (PRISMA) criteria [7]. We used an electronic search to find relevant English-language papers that investigated how the timing of surgical intervention influences patient outcomes in AC. We looked through PubMed, Web of Science, SCOPUS, and Science Direct databases. Keywords related to surgical timing and outcomes in AC were included in the search approach. Independently analyzing the search results, two reviewers chose eligible studies, retrieved data, and used appropriate assessment instruments to gauge the caliber of the included study.

Eligibility Criteria

Inclusion and exclusion criteria are illustrated in Table 1.

**Table 1:** Inclusion and exclusion criteria for the systematic review N/A: not applicable, AC: acute cholecystitis

Criterion	Inclusion	Exclusion
Population	Adult AC patients (≥18 years old)	Pediatric patients (<18 years), patients with chronic conditions significantly complicating AC
Intervention	Studies assessing outcomes based on different surgical timing (e.g., early surgery within 24-48 hours vs. delayed surgery after 48 hours)	Studies do not clearly differentiate between the timing of surgery
Outcomes	Studies reporting on clinical outcomes (e.g., complication rates, length of hospital stay, death rates, readmission rates, conversion to open surgery)	Studies focusing on guidelines without empirical data or not disclosing relevant results about the time of surgery
Study design	Cohort studies, case-control studies, randomized controlled trials	Reviews, editorials, commentary, case studies, research of inadequate methodological quality
Language	English-language articles	Studies not published in English
Time frame	Articles published within 2023-2024 (or specified relevant time frame)	N/A
Data duplication	N/A	Studies with overlapping patient populations or outcomes reported in other studies

Data Extraction

Rayyan (QCRI) was used to check the search results in order to preserve accuracy [8]. The search yielded titles and abstracts, which were then evaluated for relevance using the predetermined inclusion and exclusion criteria. The research team carefully examined all the studies that satisfied the inclusion criteria. Any disagreements were settled by consensus and conversation. Using a predetermined data extraction form, key study data were recorded, including titles, authors, publication year, study location, participant demographics, gender distribution, definitions of early intervention, definition of delayed intervention, mortality rates, complication rates, and main outcomes. An impartial evaluation instrument was developed to analyze the potential for bias in the included research.

Data Synthesis Strategy

Summary tables were made using data from relevant studies in order to provide a qualitative evaluation of the study components and conclusions. Once the data collection for the systematic review was completed, the optimal approach to using the information from the included studies was determined.

Risk-of-Bias Assessment

To assess the quality of the study, the Joanna Briggs Institute (JBI) [9] applied critical assessment criteria for research with prevalence data. There are nine questions in this tool. A good response receives a score of 1, whereas a negative, unclear, or irrelevant response receives a score of 0. The scores that followed were divided into three categories: poor, moderate, and high quality: below 4, in the range of 5 to 7, and over 8. The quality of the articles was assessed by impartial scholars, and disagreements were resolved through discussion.

Results

Systematic Search Outcomes

**Figure 1:**
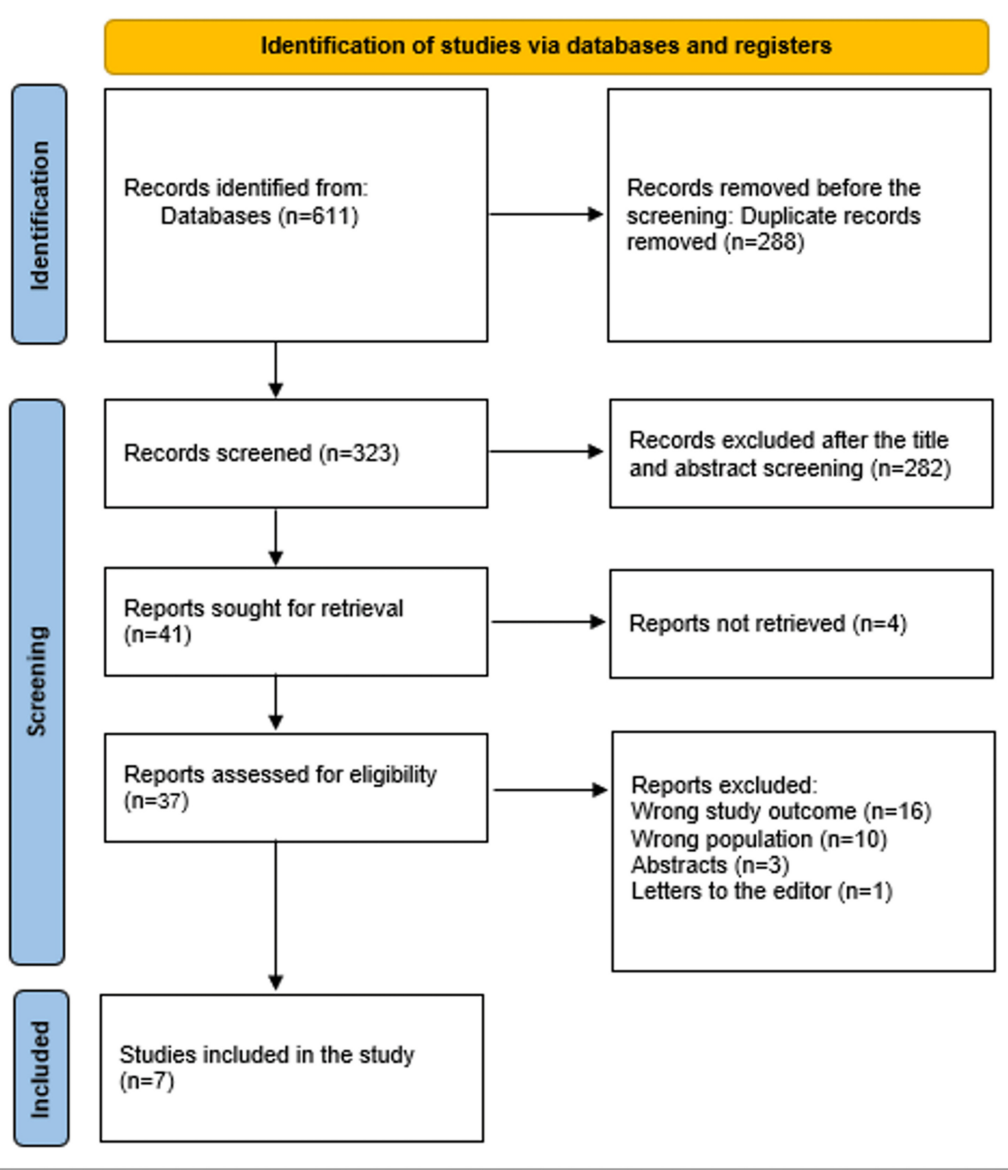
PRISMA diagram employed to encapsulate the research decisions PRISMA: Preferred Reporting Items for Systematic Reviews and Meta-Analyses

A thorough search of 611 study papers yielded 288 duplicates that were left out. After looking over the abstracts and titles of 323 studies, 282 articles were rejected. Out of the 41 reports that were necessary, four were not found. Three abstracts, one editor's letter, and the study results' inaccuracies led to the rejection of the articles. Ten of the 37 publications that passed the full-text screening stage were disqualified for using the wrong demographic types. The qualifying requirements are met by the seven research publications that comprise this systematic review. A diagram illustrates the process by which the literature was selected in Figure 1.

Sociodemographic and Clinical Parameters of the Comprised Participants and Studies

Table 2 presents the study papers' sociodemographic data. Our information comprised seven experiments with 48,747 patents: 40,955 in the early laparoscopic cholecystectomy (ELC) group and 7,792 inside the delayed laparoscopic cholecystectomy (DLC) team [10-15]. More than half of the participants (27,687, 56.8%) were females. Five studies were retrospective cohorts [4,10,11,14,15], one was an observational study [12], and one was a prospective cohort [13]. Two research projects were carried out in Australia [10,11], one in India [12], one in Turkey [4], one in Japan [13], one in China [14], and one in Romania [15].

**Table 2:** Sociodemographic parameters of the comprised studies

Study ID	Study design	Country	Participants (n)	Mean age	Females (%)
Choi et al., 2023 [10]	Retrospective cohort	Australia	459	44	169 (31.2%)
Köstenbauer et al., 2023 [11]	Retrospective cohort	Australia	47,478	66 ± 10.2	27,073 (57%)
Vidyadharan et al., 2023 12]	Observational study	India	80	40.6 ± 13.1	37 (46.2%)
Güneş et al., 2023 [4]	Retrospective cohort	Turkey	178	47.1 ± 14.4	87 (48.9%)
Kilinc Tuncer et al., 2023 [13]	Prospective cohort	Japan	92	52.8 ± 14.8	41 (44.6%)
Li et al., 2023 [14]	Retrospective cohort	China	194	46.8 ± 14.1	111 (57.2%)
Budiæcã et al., 2024 [15]	Retrospective cohort	Romania	266	57.6 ± 18.4	169 (63.5%)

Table 3 shows the clinical parameters. Early intervention, often defined as surgery performed early after the commencement of symptoms, shows promising outcomes in terms of reduced mortality and complication rates. In several studies, ELC was found to be safe regardless of when symptoms first appear, challenging previous assumptions that surgery should be delayed during the initial symptomatic period [10,12,14,15]. Mortality rates for early intervention were consistently low, and complication rates were also minimal, with mortality figures such as 0% [10,13,15] to 3.9% [11]. The complication rates ranged from 3.5% [13] to 12.5% [12].

**Table 3:** Clinical parameters and outcomes of comprised studies Def: definition, N: number, ELC: early laparoscopic cholecystectomy, LC: laparoscopic cholecystectomy, DLC: delayed laparoscopic cholecystectomy, NM: not mentioned, AC: acute cholecystitis

Study ID	Early intervention				Delayed intervention				Main outcomes	JBI
	Def	n	Mortality (%)	Complications (%)	Def	n	Mortality (%)	Complications (%)		
Choi et al., 2023 [10]	<72 hours of symptoms	305	0	12 (3.9%)	72 hours-7 days from symptoms	154	1 (0.65%)	8 (5.2%)	ELC for AC is safe no matter when symptoms first appear. It is not necessary for surgeons to restrict ELC for the first 72 hours after the start of symptoms.	Moderate
Köstenbauer et al., 2023 [11]	Within 7 days of admission	40,187	92 (0.2%)	NM	>7 days from admission	7,291	10 (0.1%)	NM	This validates the safety and effectiveness of ELC in elderly patients. When AC is present, delaying cholecystectomy is linked to worse results for the population.	Moderate
Vidyadharan et al., 2023 12]	<72 hours of symptoms	40	NM	5 (12.5%)	>72 hours from symptoms	40	NM	15 (37.5%)	ELC (less than 24 hours after biliary colic diagnosis) reduces hospital stay length and operation time.	High
Güneş et al., 2023 [4]	<24 hours of admission	90	1 (0.01%)	7 (7.7%)	>24 hours from admission	88	0	5 (5.7%)	The ELC group had the least total hospital stay duration. For the ELC and DLC groups, the postoperative hospital stays were the shortest. The longest hospital stay after surgery was recorded in the ELC group. Therefore, we do not recommend utilizing ELC.	Moderate
Kilinc Tuncer et al., 2023 [13]	<7 days of symptoms	57	0	2 (3.5%)	>7 days from symptoms	35	0	2 (5.7%)	Regarding difficult cholecystectomy and morbidity, there is no difference between early and delayed cholecystectomy for patients with grade II AC. Cholecystectomy is not facilitated by performing it during the postponed time.	Moderate
Li et al., 2023 [14]	<72 hours of symptoms	142	NM	11 (7.75)	>72 hours from symptoms	52	NM	6 (11.54)	It is not necessary to adhere to the 72-hour standard for LC, even though the state of inflammation in early cases of cholecystitis may be critical.	Moderate
Budiæcã et al., 2024 [15]	<72 hours of symptoms	134	0	14 (10.4%)	>72 hours from symptoms	132	1 (0.76%)	25 (18.9%)	For AC sufferers, ELC has a number of benefits that make it superior to DLC. Postoperative results are similar to pre-pandemic levels, even though the number of AC hospitalizations during the pandemic decreased.	High

DLC, which typically involves waiting beyond 72 hours or even seven days from symptom onset (delayed), exhibited a higher complication and mortality rate compared to early intervention. While DLC is still considered safe, the likelihood of side effects such as bile duct damage and surgical site infections seemed to increase, particularly in patients who were treated later than seven days after admission. However, some studies noted that in select populations, delayed intervention did not lead to significantly worse outcomes, especially when dealing with mild to moderate AC [4,13]. Mortality rates for DLC were consistently low, with figures such as 0% [4,13] to 0.76% [15]. The complication rates ranged from 5.2% [10] to 37.5% [12].

Discussion

The results of this systematic review provide credence to the growing body of research indicating that ELC is a secure and successful therapy for AC. Early intervention, which is defined as surgery done within 72 hours of the beginning of symptoms, was linked in most of the trials to fewer problems, shorter hospital stays, and reduced death rates. Even though it was still successful, DLC patients had a somewhat increased risk of protracted hospital admissions and surgical problems, especially if they were performed beyond seven days. Even with these conclusions, some research continues to suggest that DLC may be safe in certain populations, particularly for people with moderate instances of AC. Coccolini et al. reported that the AC needs to be run as soon as feasible. Delaying cholecystectomy until one to five weeks after first admission was the worst strategy for reducing postoperative complications; cholecystectomy should be performed no later than 72 hours after the onset of symptoms [16].

However, Gu et al. discovered that in terms of surgical complications and conversion to open surgery, there is no discernible difference between ELC and DLC while treating AC. In the clinical setting, it is worthwhile to promote the shorter surgical time and shorter hospital stay associated with ELC [17].

To ensure effective hemostasis, careful dissection of the Calot triangle and a more prolonged separation from the gallbladder bed may be necessary in cases of acute inflammation. The slight increase in operating time is likely due to the surgeon's careful consideration rather than any technological issues, as the rates of conversion to open surgery and bile duct injury are the same for both "early" and "delayed" definitions. Considering that these patients require multiple hospital stays to finish their treatment, it is logical that the observation that "delayed" definitions led to a longer overall hospital stay [7].

We found that mortality and complication rates for early intervention were consistently lower than for delayed intervention. In contrast, Wang et al. found that the ≥2-week group has a lower risk of less tissue damage, quicker recuperation, improved healing, and reduced intraoperative blood loss when treating AC. After two weeks, it is evident that LC is secure and beneficial for AC sufferers [18].

The findings show that surgeons should prioritize ELC wherever possible because of the many advantages it offers, including fewer issues, shorter hospital stays, and faster overall recovery durations. Given the evident benefits of early intervention, current clinical guidelines may need to be updated to reflect these findings. Surgeons should be trained on the safety of conducting ELC within 72 hours of symptom onset, even in patients with advanced inflammation. Implementing early intervention protocols may lower hospital expenditures related to prolonged hospitalizations and complications from delayed surgery. By lowering the amount of time spent in the hospital, resources may be deployed more efficiently, resulting in better patient outcomes and reduced healthcare expenditures.

Limitations

Despite the optimistic results, the research evaluated has major limitations. For starters, many studies are observational, and while they provide useful information, they lack the rigor of randomized controlled trials. Some studies also have small sample numbers or are conducted in single-center settings, which can restrict the generalizability of the findings. Furthermore, the definitions of early and delayed intervention vary among studies, with some utilizing different cut-off points for "early" surgery. Because of the absence of consistency, comparing results across studies is challenging. Another drawback is a lack of precise data on specific patient characteristics, including comorbidities and preoperative risk factors, which may influence results. Finally, the trials are mostly concerned with short-term outcomes, with little attention placed on long-term follow-up, notably for symptom recurrence or long-term consequences.

## Conclusions

ELC within the first two days is a safe and effective treatment for AC when compared to DLC, providing better outcomes. However, there is a noticeable rise in risk-adjusted mortality beginning on the third day of acute symptoms. ELC is associated with lower complication rates, reduced hospital stays, and minimal mortality, suggesting it should be the preferred approach in most cases. While delayed intervention remains a viable option, particularly for certain patient populations, it carries a higher risk of complications and prolonged recovery times. Further research, particularly randomized controlled trials, is needed to confirm these benefits and refine surgical timing strategies for different patient groups.
